# Periprosthetic Joint Infection With *Salmonella* Species 2 Years After Unicompartmental Knee Arthroplasty Treated With Debridement, Antibiotics, and Implant Retention (DAIR): A Case Report and Review of the Literature

**DOI:** 10.1155/cro/2056500

**Published:** 2026-04-15

**Authors:** Panagiotis Antzoulas, Vasileios Giannatos, Christos Michailides, Maria Lagadinou, Andreas Panagopoulos, Evangelia Argyropoulou, Vasileios Athanasiou, John Gliatis

**Affiliations:** ^1^ Department of Orthopedics and Traumatology, University Hospital of Patras, Patras, Greece, pgnp.gr; ^2^ Medical School, University of Patras, Patras, Greece, upatras.gr; ^3^ Infectious Disease Department, University Hospital of Patras, Patras, Greece, pgnp.gr

**Keywords:** DAIR, periprosthetic joint infection, PJI, *Salmonella*, UKA

## Abstract

**Background:**

Unicompartmental knee arthroplasty (UKA) is a minimally invasive surgical procedure aimed at treating selected patients with osteoarthritis confined to a single compartment of the knee. The concept is to minimize soft‐tissue damage retaining the natural ligaments and thus providing kinematics close to normal knee. Periprosthetic joint infection (PJI) is a potentially devastating complication following UKA. Although not a common occurrence, it can lead to prolonged hospitalization, need for repeated surgical intervention, and joint failure. Although *Staphylococcus* spp. consisting the majority of the pathogens, *Salmonella* can also be found on rare occasions, particularly in individuals with immunosuppression or vascular disease in the setting of underlying diseases such as sickle cell disease, diabetes mellitus, renal failure, human immunodeficiency virus (HIV) infection, or chronic corticosteroid use.

**Case Report:**

We describe a case of PJI of the knee following UKA in a 73‐year‐old woman with a history of long‐term corticosteroid oral treatment for polymyalgia rheumatica (PMR). The patient presented with a painful, swollen right knee and elevated inflammatory markers. She was successfully treated with arthroscopic drainage, irrigation, and a 9‐week course of antibiotic therapy.

**Conclusion:**

This case highlights the virulence of *Salmonella* in an immunocompromised patient with a joint prosthesis. To our knowledge, this is a rare case that has not previously been reported in the literature. Continuous monitoring, early diagnosis, and close collaboration between infectious disease specialists and orthopedic surgeons were crucial in achieving a successful resolution of the infection.

## 1. Introduction

Unicompartmental knee arthroplasty (UKA) is a surgical option designed to replace osteoarthritis confined to a single compartment. It is an increasingly popular surgical procedure that provides benefits such as quicker recovery and preservation of native structures compared with total knee arthroplasty (TKA) [1]. For seven years, from 1998 to 2005, the number of UKAs performed in the United States saw a significant rise, increasing almost eightfold [2]. In the United States, Australia, England and New Zealand according to their national joint replacement registries, UKA usage is reported at 2%–12% in clinical practice [3–6]. Patients undergoing UKA often experience significant improvements in knee function, reduced pain, and quicker recovery times, which have led to the growing popularity of the procedure [7], [8].

Periprosthetic joint infection (PJI) is a serious and potentially devastating complication following joint replacement surgery. Infections can lead to significant morbidity and often require complex surgical and medical management [7]. PJI is a well‐known complication following total TKA, occurring in approximately 1%–2% of cases [9], [10]. However, the incidence of PJI is notably lower in UKA, with reported rates ranging from 0.1% to 0.8% [11].

UKA is generally a safe procedure in terms of PJI complications, with incidence below 1.5%, suggesting a lower risk of infection compared with TKA. However, PJI epidemiology and microbiology are not yet well described in UKA [12–15].

We describe a case of PJI following a UKA in a 73‐year‐old woman with a history of long‐term corticosteroid treatment for polymyalgia rheumatica (PMR). To our knowledge, this is the first case of *Salmonella* spp. infection of UKA described in the literature.

## 2. Case Report

A 73‐year‐old woman who had undergone a mobile‐bearing cemented UKA (cemented Oxford Partial Knee, Zimmer‐Biomet, Warsaw, Indiana, United States) on her right knee 2 years ago (Figures 1 and 2), presented to the emergency department with pain and swelling in her right knee for the last 8 days, without any history of trauma (Figure 3). Written consent for publication was acquired from the patient. Her clinical presentation was deteriorating, reporting a fever up to 38.9°C during the last 2 days. She mentioned a medical history of PMR, for which she was treated with 4 mg of methylprednisolone tablet daily, as well as hypertension, osteopenia, hypothyroidism, depression, chronic obstructive pulmonary disease (COPD), and dyslipidemia. At presentation in the emergency department, her temperature was 39.3°C, oxygen saturation was 94%, and blood pressure was 165/94 mmHg. Blood tests showed elevated inflammatory markers: WBC 11.47 K/*μ*l, lymphocytes 85.2%, monocytes 11.2%, platelets 221 K/*μ*l, CRP 20.16 mg/dL, and ESR 110 mm/h. The COVID‐19 antigen test was negative, and a triplex ultrasound ruled out deep vein thrombosis in her right lower limb.

Figure 1Preoperative x‐rays (a) anteroposterior and (b) lateral, taken before the UKA revealed that the patient had osteoarthritis localized to the medial compartment of the knee.

**Figure:**
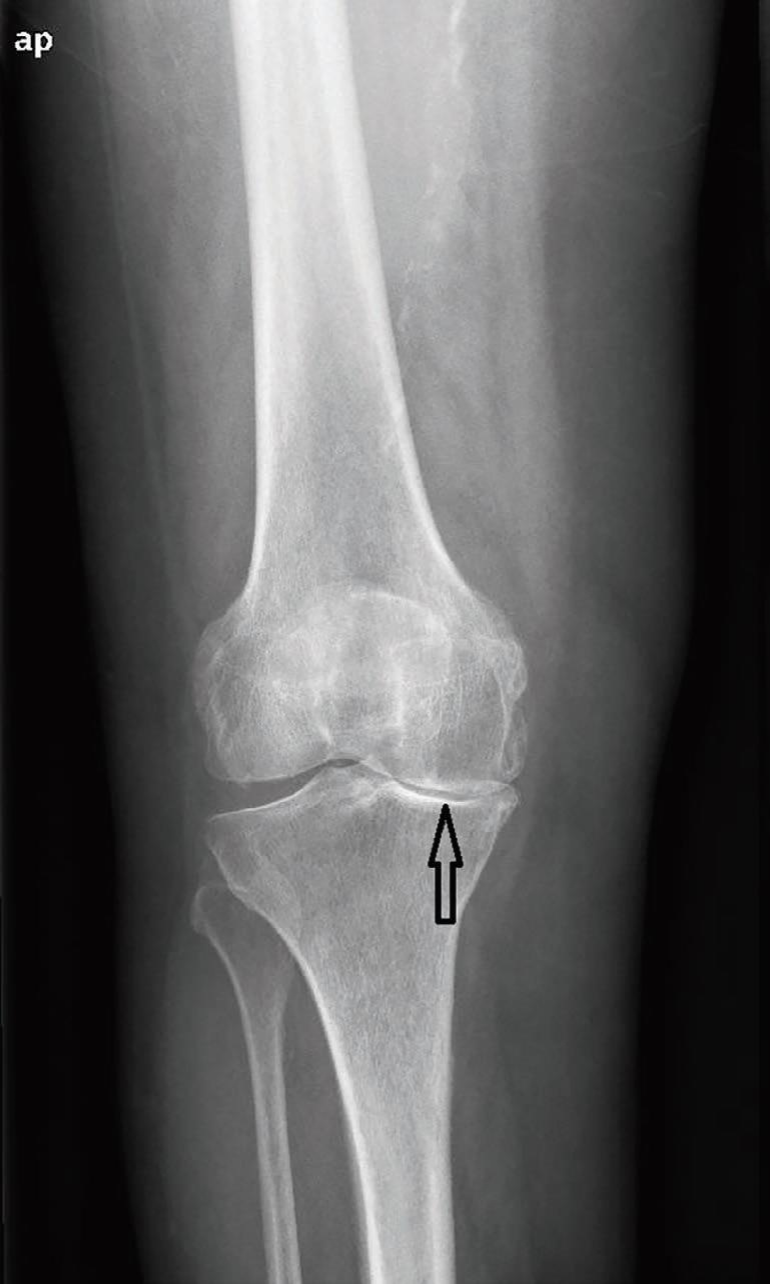
(a)

**Figure:**
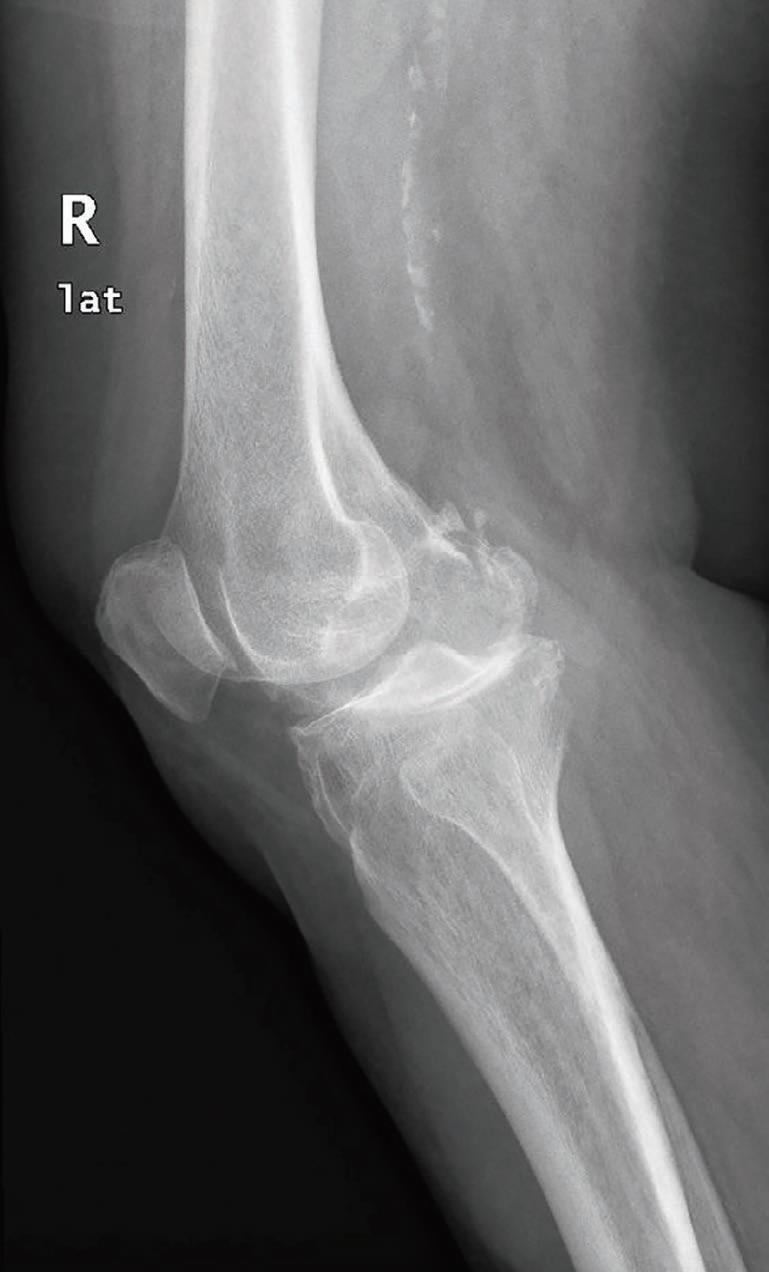
(b)

Figure 2X‐rays (a) anteroposterior and (b) lateral, taken 1 day after the initial surgery confirmed the placement of the UKA.

**Figure:**
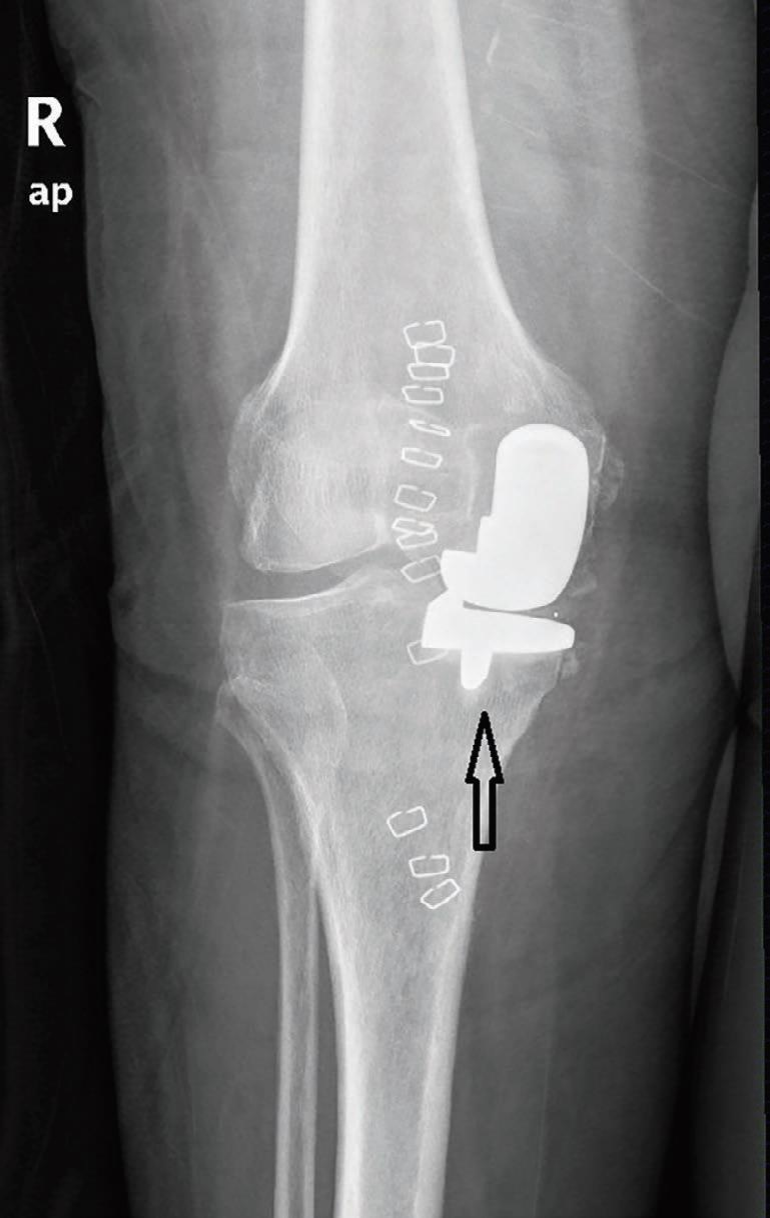
(a)

**Figure:**
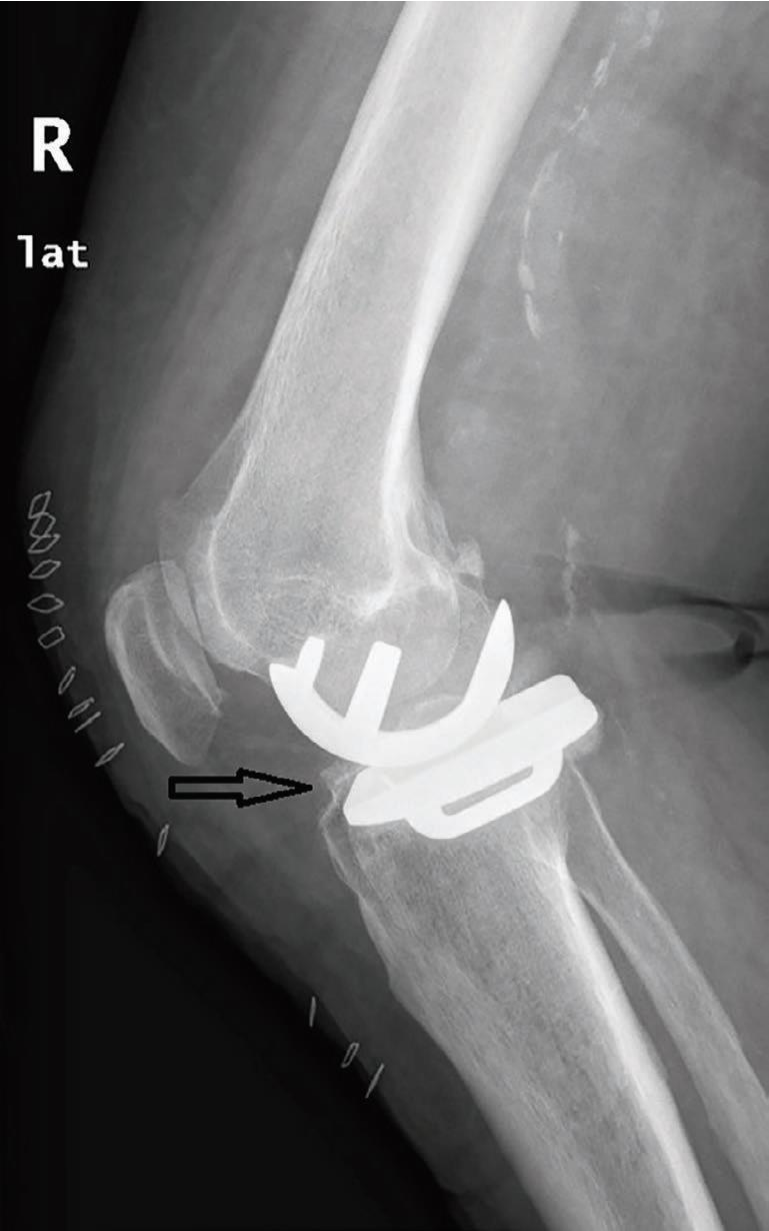
(b)

Figure 3X‐rays (a) anteroposterior and (b) lateral, were performed when the patient presented to the emergency department with inflammation, which was later associated with salmonella infection, revealing no radiological signs of implant loosening.

**Figure:**
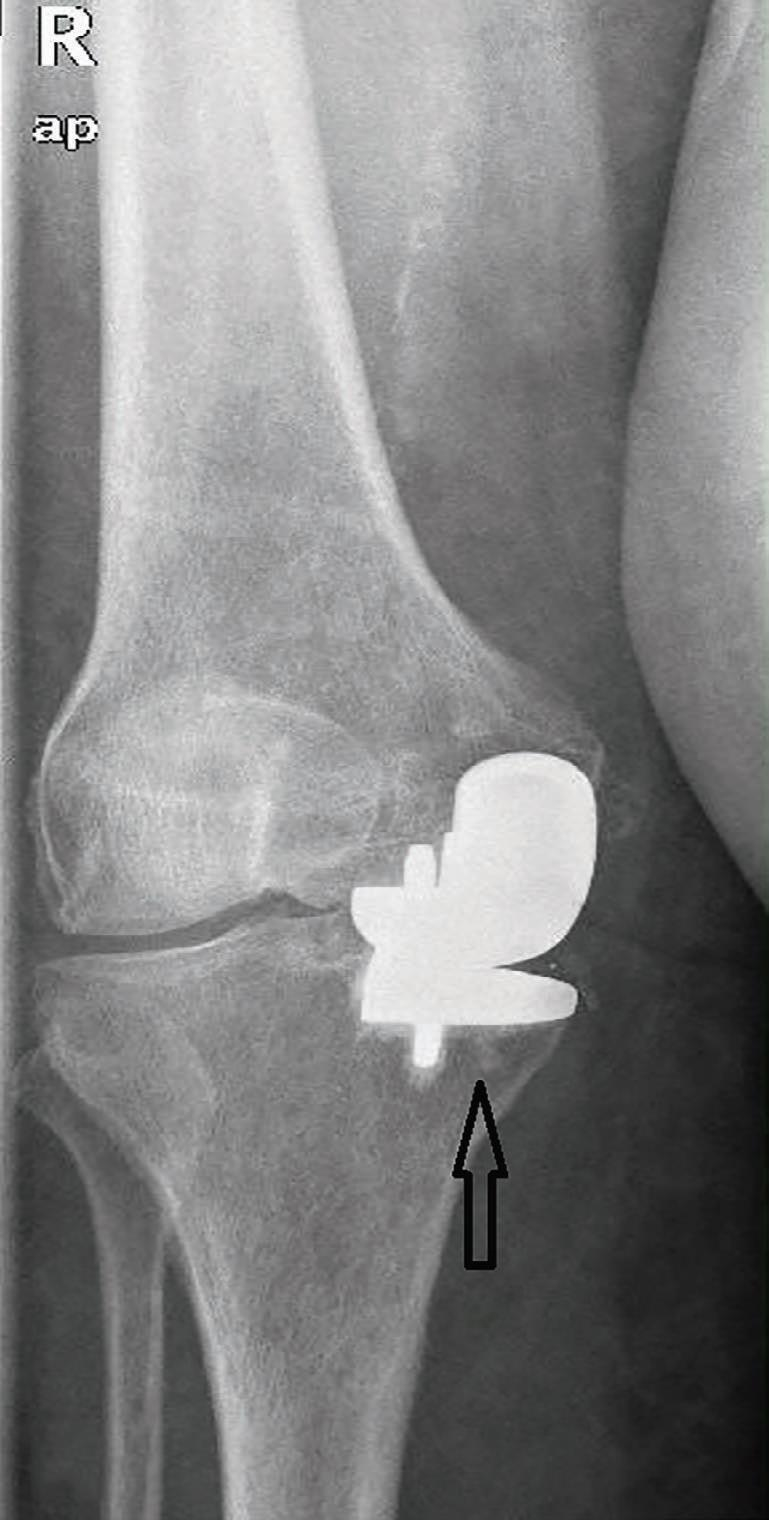
(a)

**Figure:**
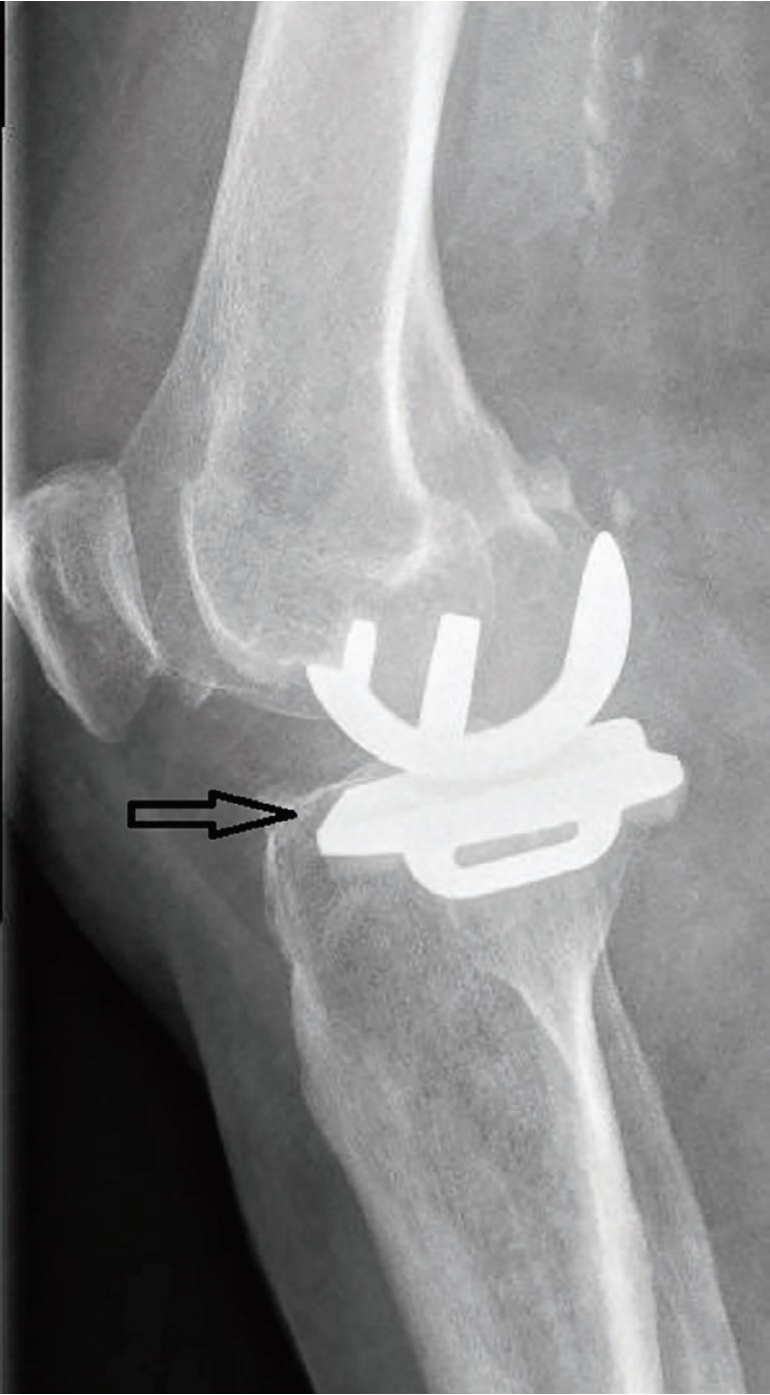
(b)

We aspirated 60 mL of cloudy, purulent fluid from her joint. The synovial fluid analysis revealed a WBC count of 206,400 cells/*μ*L, consisting of 94% polymorphonuclear cells, and a negative Gram stain, suggesting Gram‐negative bacteria. The patient was started on intravenous (IV) piperacillin–tazobactam (4 + 0.5g qid) and vancomycin (1 g bid).

**Figure 4:**
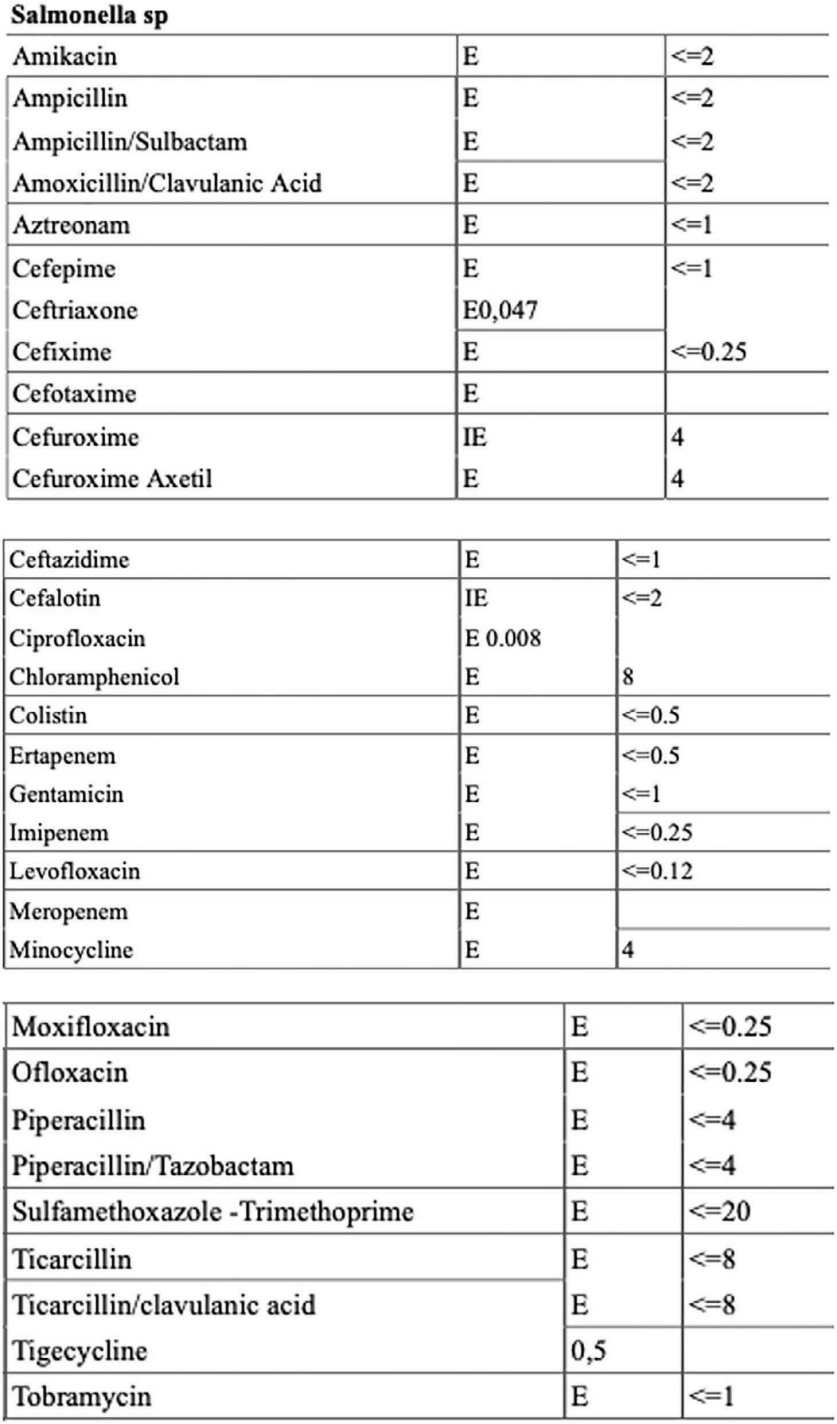
Blood culture and antibiogram identified *Salmonella* species, a Gram‐negative bacterium, with the following results: sensitive (E) and intermediate (I) to the tested antibiotics.

The following day, the patient underwent arthroscopic drainage and irrigation of the joint. Arthroscopic debridement is a technique‐dependent modality that needs careful execution with thorough synovectomy of the infected tissue and joint lavage with normal saline intraoperatively. An anteromedial and anterolateral portal were used with 6 L of saline and a motorized shaver for synovectomy. Based on the infectious disease specialist′s advice, vancomycin was discontinued, and daptomycin 750 mg IV once daily was initiated. After 3 days, joint fluid cultures grew *Salmonella* spp., confirming the Gram‐negative infection (Figure 4). Urine and blood cultures were negative. After completing 12 days of treatment, the patient was afebrile with negative CRP level. The infectious disease specialist discontinued the IV antibiotics and transitioned the patient to oral ciprofloxacin (500 mg bid) for another 7 weeks.

She underwent weekly blood tests to monitor CRP, WBC, urea, and creatinine levels. The rehabilitation protocol following septic arthritis after UKA was individualized based on the patient′s condition and progress. Early passive ROM and adequate analgesia were sought during the acute phase, followed by partial weight bearing utilizing forearm crutches during Weeks 0–2 and full weight bearing along with functional rehabilitation and strengthening exercises thereafter. During the rehabilitation, good hygiene practices and management of medical comorbidities were enthusiastically endorsed to prevent a relapse. A multidisciplinary approach, including input and communication between orthopedic surgeons and infectious disease specialists is crucial for optimizing recovery and preventing devastating complications. The incorporation of nursing practitioners in the team and physical therapists is also vital for close patient monitoring as well as full functional rehabilitation and return to daily living. At her 2‐year follow‐up, the patient remained healthy without any signs of infection and she did not require revision surgery or further antibiotic therapy (Figure 5). The clinical outcome was evaluated using the International Knee Society (IKS) scores, which confirmed a successful recovery.

Figure 5X‐rays (a) anteroposterior and (b) lateral, taken 2 years after the Salmonella infection showed the long‐term effect.

**Figure:**
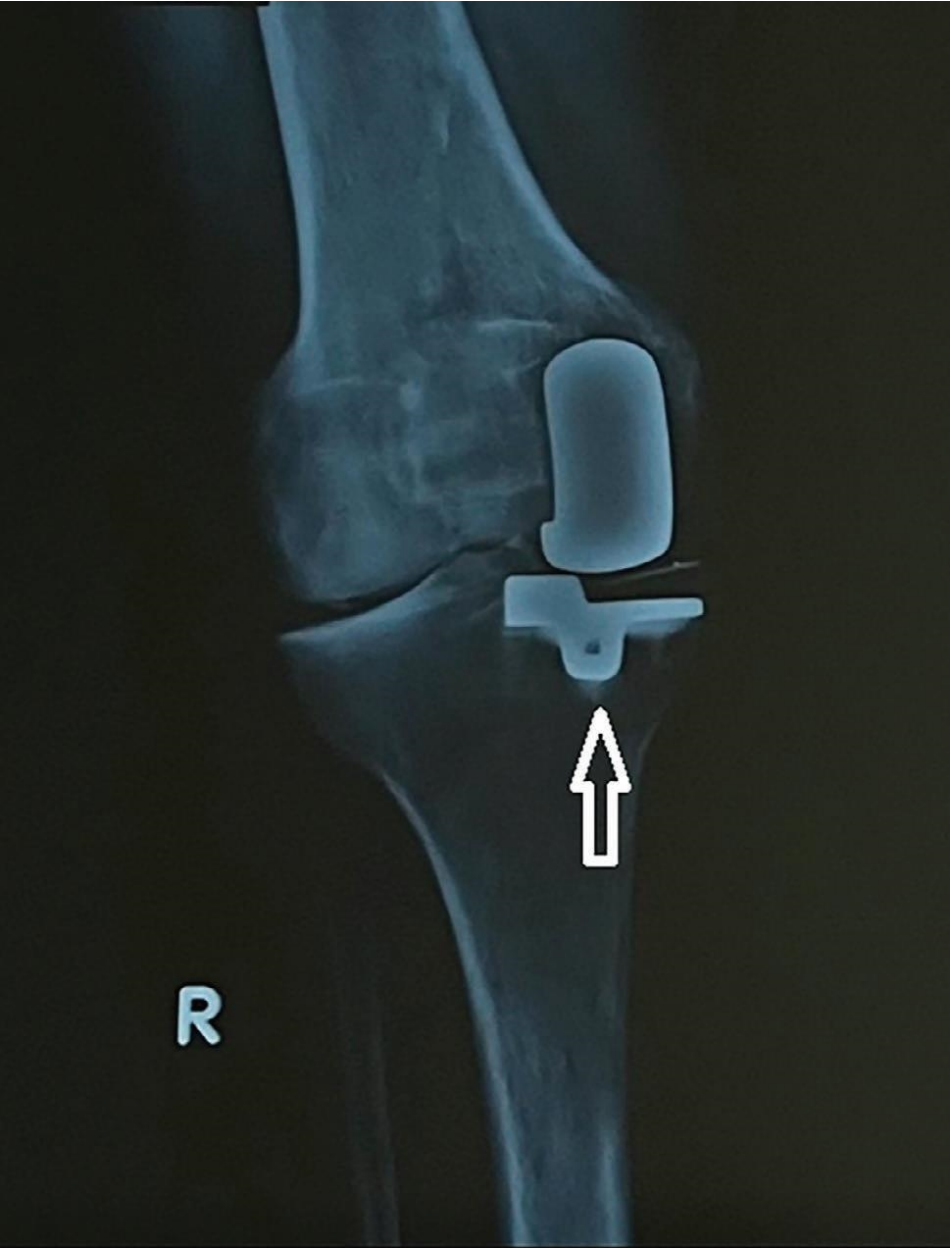
(a)

**Figure:**
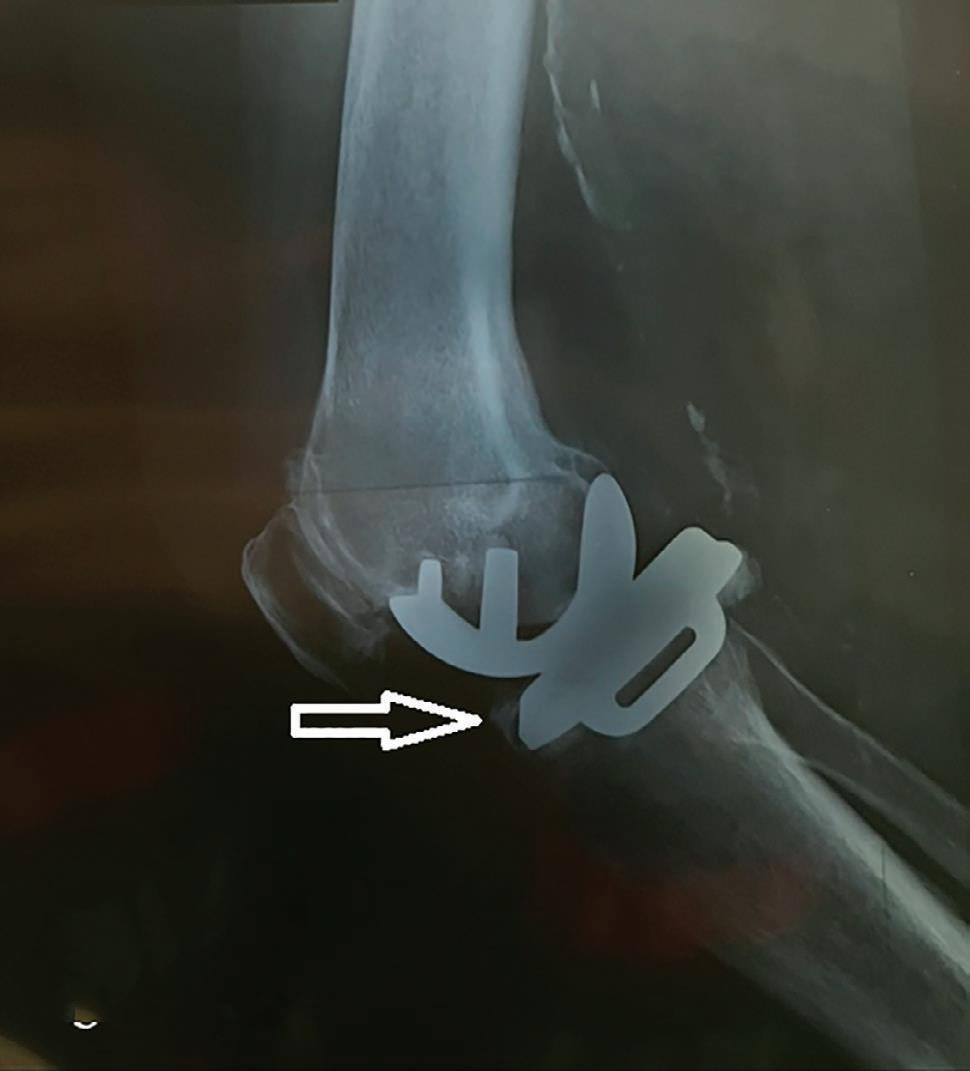
(b)

## 3. Discussion

This is the first case of *Salmonella* spp. infection of UKA described in the literature. This pathogen is considered rare even in TKAs and only a small number of cases have been previously reported (Table 1). *Gram-negative* PJIs are rare compared with *Gram-positive* infections so *Salmonella* spp. is considered an atypical microorganism for this site [23–26]. *Escherichia coli* and *Pseudomonas* spp. are the most common *Gram-negative* pathogens for PJIs [12]. The clinical significance of this is that certain *Gram-negative* pathogens can elude diagnosis, as traditional synovial fluid or deep tissue cultures underreport their presence, although modern techniques, such as metagenomic next‐generation sequencing (mNGS) or sonication of the prosthetics, perform fairly well. Hao et al. demonstrated that *Pseudomonas* and *Serratia* spp. were only detected by mNGS in synovial fluid, in contrast to *Klebsiella* spp.*, E*. *coli, anaerobes, Gram-positive,* and *fungi* [13]. Fang et al. also demonstrated that enhancing blood cultures with mNGS can drastically increase the sensitivity, accuracy, and specificity [14].

**Table 1:** *Salmonella* spp. knee arthroplasty infection.

Title	Author (year)	Patient Age (years)	Risk factors	Medications	Microorganism	Site of infection	Time onset since surgery	Treatment
Bilateral periprosthetic infection caused by *Salmonella enterica* serotype Enteritidis and identification of *Salmonella* sp. using molecular techniques	Kobayashi et al. [16]	71	RA	prednisone: 5 mg qd, AZA: 100 mg qd, HCQ: 400 mg qd, MTX: 15 mg/week	*Salmonella enterica*	Bilateral TKA	6 and 11 years, respectively	Ciprofloxacin, undetermined IV, PO duration
Salmonella septic arthritis following total knee arthroplasty for rheumatoid arthritis in a patient receiving etanercept	Oe et al. [17]	61	RA	methylprednisolone, etanercept (last 2 weeks before surgery), MTX	*Salmonella enteritidis*	TKA	5 weeks	2 weeks IV meropenem, 2 weeks PO levofloxacin, 3 months PO minocycline
*Salmonella enterica* arthritis in a patient with rheumatoid arthritis receiving antitumour necrosis factor therapy	Bubonja‐Sonje et al. [18]	62	RA	prednizolone: 5 mg qd, MTX: 15 mg/week, infliximab	*Salmonella enteritidis*	TKA	3 years	Exchange arthroplasty, IV, PO treatment undetermined
*Salmonella* Typhimurium arthritis in rheumatoid disease	Rae et al. [19]	67	RA	prednizolone: 7.5 mg qd, penicilamine: 125 mg qd	*Salmonella enterica*	TKA	5 years	2 weeks IV chloramphenicol 1 g/d and IA gentamycin 80 mg qd, then ampiciline 1 g qid IV, PO duration undetermined
*Salmonella enteritidis* infection in total knee replacement	Madan et al. [20]	75	RA	Undetermined corticosteroids	*Salmonella enteritidis*	TKA	8 years	6 months PO ciprofloxacin 500 mg bid followed by another 3 months recurrence
Salmonella prosthetic joint septic arthritis	Day et al. [21]	55	OA	Not reported	*Salmonella enteritidis*	TKA	12 days	6 weeks IV ceftriaxone 2 g/week
Salmonella infection in joint arthroplasty	Musante et al. [22]	35	OA	Not reported	*Salmonella enterica*	TKA	8 weeks	Not reported

Abbreviations: AZA, azathioprine; HCQ, hydroxychloroquine; IA, intra‐articular; IV, intravenous; MTX, methotrexate; OA, osteoarthritis; PO, per os; RA, rheumatoid arthritis; TKA, total knee arthroplasty.

PJIs in UKAs are not yet well reported. Data are derived mainly from some retrospective observational studies that analyzed databases of patients who had undergone UKA [15, 23–27]. Agarwal et al. calculated an adjusted odds ratio (aOR) of 0.868 for infection in favor of UKA compared with TKA [15]. Yamagami et al. described a statistically significantly lower incidence of UKA PJI compared with TKA (0.3% vs. 0.6%, OR 0.47) [23]. In a wide database screening of 32379 UKAs and 250377 TKAs in US and UK hospitals a hazard ratio of 0.56 for PJI slightly favored UKAs [24]. Brivio et al. found 3225 cases of UKA of which 19 complicated with PJI and were treated with DAIR. A total of 3/19 had recurrent infection and underwent a second DAIR, median 20 days later. A total of 17/19 had *Gram-positive* infection, one of them grew *Acinetobacter* spp. in fluid culture and another one grew *Enterobacter* spp. A total of 2/17 *Gram-positive* and the one with the *Enterobacter* spp. PJI suffered from recurrent infection. All patients were treated with a 2‐week IV antibiotic course, followed by 6 weeks of oral treatment and the survivorship of all‐cause reoperation was approximately 79% [25]. Kocaoğlu et al. reported 15 infected UKAs, all due to *Gram-positive* microorganisms. One of them was reinfected by *Citrobacter* spp. and was amputated. Survival rate at 3‐year follow up was 9/15 and the overall survival free of any reoperation was 80% at 5 years [26]. Cavagnaro et al. described 16 infected UKAs. A total of 3/16 were culture negative. Among the remaining 13, 3/13 were polymicrobial infections, 10/13 had *Gram-positive* microorganisms, and 4/13 had *Gram-negative* microorganisms. After two‐stage revision, infection free survivorship was 100% [27]. Despite fluid culture being reliable, in some cases diagnosis of PJI can be challenging. mNGS seems to perform better for Gram‐negative PJIs and the combination of synovial nucleated cells, polymorphonuclear cells, and serum CRP has excellent diagnostic accuracy for UKA PJI (AUC 0.97). ESR can also assist diagnosis in UKA PJI (AUC 0.89) [13], [28].

In our case, after a short duration of IV antibiotic treatment, a switch to active oral agents against detected microorganism was implemented. That was in line with the results of a randomized controlled trial performed by Manning et al. that demonstrated noninferiority of the 2‐week IV course followed by oral switch compared with the 6‐week IV course [29].

In our case, the patient suffered from PMR and was under oral corticosteroid (CS) treatment. There is evidence that cortisone injections or CS treatment may increase the incidence of PJI after knee replacement. Three meta‐analyses agree that CS intra‐articular injection within 3 months of knee arthroplasty increases the risk of PJI but one meta‐analysis found no significantly increased risk [30–33]. Our patient was treated with 4 mg of methylprednisolone tb per os daily for her PMR. A retrospective study by Piple et al. demonstrated that patients on prednisone that underwent TKA or total hip arthroplasty (THA) had increased risk of PJI compared with those who did not receive CS, in a dose dependent manner (1.17% vs 0.52%, aOR 2.14overall, 1.64 for 0–5 mg, 3.09 for > 30 mg) [34]. In a systematic review OA as indication for TKA is described to have lower risk of PJI compared with RA for TKA with an OR of 2.04 [35].

Only seven cases of *Salmonella* spp. PJI are reported in the literature, all of them in patients who had undergone TKA [16–22]. A total of 5/7 were RA patients and 2/7 were OA patients. All RA patients were under immunosuppressive agents including CS, but for the two OA patients treatments are not reported. Time since TKA varied from 12 days to 11 years. A total of 3/7 had *Salmonella enterica* infection and 4/7 had *Salmonella enteritidis* infection. Duration and regimen of PO and IV treatment also varied among those patients. Table 1 summarizes the characteristics of those cases. On the other hand, a 2025 case‐control study by Nazligül et al. demonstrated that despite a diverse microbiological profile in TKA PJI, UKA presented only with Gram‐positive *Staphylococcus* [36].

Particularly interesting is the fact that DAIR procedure was enough in our case and no revision of the implants was needed. Oe et al. reported a success rate of 27%–40% for implant retention after PJI with *Salmonella* spp. [17]. Despite the incidence occurring 2 years after the operation, prompt diagnosis after symptom onset and immediate DAIR procedure (8 days after symptoms′ onset) might have played a decisive factor for success in our case. A favorable resistance profile of the pathogen might have also contributed to the successful management, since extensive fluoroquinolone resistance has been reported among *Salmonella* spp. in the last decades, but in our case ciprofloxacin (MIC = 0.008) was enough to treat the pathogen [37]. Finally, the nature of the UKA implant might have also impacted the favorable outcome, in comparison with the known principles for TKA PJI treatment. All of the above allowed us to successfully treat this PJI case using not only DAIR alone, but also an arthroscopic minimally invasive version of it. We believe that being extra careful to perform an arthroscopic synovectomy also enhanced the outcomes of this procedure.

## 4. Conclusion

This case highlights a successful treatment of a late infection with *Salmonella* spp. after knee UKA with DAIR. Although not the gold standard for late infections, prompt diagnosis from early symptoms′ onset and close multidisciplinary approach can lead to a successful outcome with a thorough arthroscopic synovectomy and antibiotics. High suspicion must be maintained for atypical pathogens in immunocompromised patients.

## Funding

The publication of this article in OA mode was financially supported by HEAL‐Link.

## Consent

Patient consent was acquired for case publication.

## Conflicts of Interest

The authors declare no conflicts of interest.

## Data Availability

The data that support the findings of this study are available from the corresponding author upon reasonable request.
